# A Case of Reproduction of the Inferior Maxilla after Necrosis from Phosphorus Vapor

**Published:** 1851-04

**Authors:** 


### ARTICLE XI.

### *A Case of Reproduction of the Inferior Maxilla after Necrosis from Phosphorus Vapor.*

In the summer of 1847, Professor Virchow was requested, by staff-surgeon Sachs, to make an examination of the body of a young girl who had recently died of disease of the lungs, and who had about nine months before lost the left half of her lower jaw by phosphoric necrosis. On examining the state of the maxilla, Hr. Virchow was greatly surprised to find a firm, strong, unusually curved bone, intimately united anteriorly with the sound half of the jaw, and movable at the joint—in short, a complete regeneration of the bone. The original necrosed portion of bone was found in its former position: it had given rise to profuse suppuration, and had become so nearly detached from its connections, that it only required division in the middle to be removed with ease. It consisted of the entire half of the lower jaw, with seven alveolar cavities, the angle, the articulating process, and the greater part of the coronary process. It weighed, (with a few detached exostoses,) 21.790 grammes, (=nearly 5½ drachms Eng.,) and measured, from the lower border of its angle to the point of separation anteriorly, 3⅛ inches. The anterior portion exhibited, both on its anterior and posterior surface, irregular cavities on its surface, which, in many parts, was completely destroyed. The canals for the passage of the vessels, especially on the internal border of the alveolar processes, were much enlarged. The posterior surface of the bone, from the posterior foramen along the extent of the molar teeth, presented the appearance of pumice-stone—a dirty grey color, and its surface was studded with small exostoses.

The new bone exhibited the outward form of the necrosed portion. The body was irregularly round. The alveolar processes replaced by a flat surface, pierced by a canal for the vessels. Along its lower border to its angle it measured 3¾

inches. It was sharply curved anteriorly. The angle of the bone was of the same shape in both, only that it was more acute in the new bone. The length of the processes corresponded. Both the processes terminated in small points, to which were attached stout ligamentous bands, by which the bone was connected to the adjacent parts: a true joint had not been formed. Above the angle, in the middle of the ramus, was a circular aperture half an inch in diameter. It was filled up by yellow fibrous tissue, which united at this spot the two layers of periosteum, and through which numerous vessels passed, to be distributed through grooves or canals in the bone. This bone weighed 37.820 grammes, (between 9 and 10 drachms Eng.;) it had a reddish color, and a slight spongy tissue.

Hr. Virchow appends some remarks to his relation of this case, pointing out that the disease should rather be regarded as periostitis than necrosis, and, referring to notices of other cases of regeneration of the inferior maxilla, to prove that the same may obtain, as is found in the case of other bones.—*Verhandlungen Physicalisch-Medicinisch Gesellschaft in Wüzburg*, 1850.

